# Combination Therapeutic Effect of Antibacterial and Antiviral Agents on Feline Chronic Gingivostomatitis Nonbounded to Prior Tooth Extraction Confirmed by Physical Signs and Clinical Biomarkers

**DOI:** 10.3390/vetsci13040363

**Published:** 2026-04-08

**Authors:** Masato Katayama, Yukina Uemura

**Affiliations:** Bloom Animal Hospital, Kajiyama 1-10-32, Tsurumi, Yokohama City 230-0072, Japan; marble1993.22@gmail.com

**Keywords:** feline, chronic gingivostomatitis, antibacterial, antiviral, clinical biomarker, tooth extraction

## Abstract

Background: Feline chronic gingivostomatitis (FCGS) is refractory stomatitis in cats. The cure rate of tooth extraction selected as a primary surgical treatment is insufficient. Methods: 52 FCGS-suspected cats, including 22 tooth-extracted and 30 unextracted cats, were studied. Commercially available antiviral and antibacterial agents were orally administered as initial treatment, followed by the antiviral agent solely as maintenance therapy. We examined the influence of prior tooth extraction on the therapeutic efficacy of these two drugs by analyzing changes in some physical signs and clinical laboratory biomarkers. Results: Mass spectrometric analysis revealed the active ingredients of antibacterial and antiviral were Moxifloxacin and Molnupiravir, respectively. All physical signs (weight, appetite, activity level, grooming behavior, ptyalism, erythema) showed statistically significant improvements with combined administration of these drugs. Numbers of white blood cells, neutrophils and monocytes, as well as circulating levels of total protein, albumin, globulin, and serum amyloid-A all statistically significantly decreased with their administration (*p* < 0.0001). Conclusions: No statistically significant differences were detected between two FCGS groups in the changes in any of the above physical signs or clinical biomarker levels, indicating combination therapy with antibacterial and antiviral agents leads to effective treatment of FCGS, regardless of whether prior tooth extraction was performed or not.

## Simple Summary

The efficacy of combination therapy with an antibacterial and an antiviral agent in cats with feline chronic gingivostomatitis was demonstrated in this study. Its therapeutic effect could be confirmed by determining circulating levels of multiple hematological parameters and usual clinical laboratory biomarkers, and also easily assessed by evaluating their physical signs. Because the efficacy of this treatment was similar between with and without tooth extraction, this combination therapy can be understood to be preferable as compared with surgical tooth extraction.

## 1. Introduction

Feline chronic stomatitis is a chronic ulcerative disease affecting up to 26% of domestic cats. There is no significant difference when their ages at disease onset, sex, or breed were compared to data from controls [1,2,3]. When inflammation occurs in the caudal portion of the oral cavity (near the throat), it is designated as feline chronic gingivostomatitis (FCGS), and symmetric inflammation is observed in the mucosa near the throat [3]. Severe FCGS generally significantly reduces the quality of life of cats and is sometimes referred to as intractable stomatitis. Such an inflammatory syndrome can cause bleeding and severe oral pain, leading to dysphagia, anorexia, decreased grooming behavior and weight loss, potentially resulting in withdrawn behavior and significant stress for cats [3,4,5].

Nearly all FCGS cases present with moderate to severe periodontal disease and tooth resorption. Surgical removal of the periodontal inflammatory lesions through tooth extraction can significantly improve symptoms or even completely cure some cats [6]. Therefore, surgical treatment, including tooth extraction, has generally been considered the first-line treatment for FCGS. However, a recent review of the efficacy of surgical removal of the periodontal inflammatory lesions, including tooth extraction, revealed a 39% success rate and a 28% remission rate, suggesting that surgical removal alone has limitations in treating FCGS [3,4]. Currently, medical treatment, such as the administration of antibiotics, anti-inflammatory drugs, and analgesics after tooth extraction, is increasingly emphasized [6].

In addition, FCGS cases that do not show symptomatic improvement after tooth extraction have been reported to frequently be infected with certain viruses and bacteria. Viruses such as feline calicivirus (FCV), feline herpesvirus type 1 (FHV-1), feline immunodeficiency virus (FIV), and feline leukemia virus (FeLV) have been implicated, with FCV being particularly prevalent [4,5,7]. Chronic bacterial infections, including zoonotic oral resident bacteria such as *Pasteurella multocida*, *Porphyromonas* species, and periodontal disease-associated bacteria such as *Fusobacterium nucleatum*, have also been frequently detected [5,8]. It has been suggested that host immune response associated with diversity of the oral microbiota may be a major cause of FCGS [3,9].

Based on these previous clinical findings and case study reports, we have hypothesized that a combination of a broad-spectrum antibacterial agent and an antiviral agent effective against the above-mentioned adventitious infectious agent species might be effective in treating FCGS. Therefore, we investigated the efficacy of formulation characterized by the stepwise administration of these two components, either as a combination drug or as a single agent, by examining the temporal changes in various biomarkers (physical signs and clinical laboratory parameters) in the cats during the administration period. We have also investigated whether statistically significant differences were detected in the trends in these biomarker levels between the two groups with and without prior tooth extraction and examined whether tooth extraction affected the efficacy of this combination therapy with antibacterial and antiviral agents.

## 2. Materials and Methods

### 2.1. Drugs and Administration

The first of the orally administered drugs was Mutoral^®^I tablet (Mutoral-I), containing antibacterial and antiviral agents, and the second one was Mutoral^®^II tablet (Mutoral-II) as a single antiviral agent. Both were commercially available from Mutian Life Sciences Bio Co., Ltd. (Nantong, China). Oral administration of these formulations to FCGS cats was performed basically according to the manufacturer’s recommendations. Briefly, Mutoral-I was administered orally to cats at a dose of 1/2 tablet per kg of body weight every 12 h. Treatment continued for 2 or 4 weeks until improvement in the disease condition was confirmed; only one of the 52 cats received treatment for more than 4 weeks (44 days). After completion of Mutoral-I treatment, Mutoral-II was administered orally at a dose of 1/4 tablet per kg of body weight every 24 h for a minimum of 45 days and a maximum of 10 weeks. All oral administrations were performed by the cat owners under the guidance of the veterinarians.

### 2.2. Mass Spectrometric Analysis of Active Ingredients

Manufacturer’s information regarding the active ingredients of Mutoral-I and II formulations is suggested to be partially unclear [10]. For this reason, we performed mass spectrometric analysis of them in ethanol extracts from Mutoral-I and II tablets. We purchased commercially available Moxifloxacin (CAS No. 354812-41-2) and Molnupiravir (CAS No. 2349386-89-4) (Catalog No. S5535 and No. S8969, Selleckchem, Houston, TX, USA) as reference materials to confirm the active ingredients of each formulation.

The tablets were crushed into powder inside the package, transferred to a 2 mL tube, and dissolved in 300 µL of ethanol. An equal volume of the tablet solution and α-Cyano-4-hydroxycinnamic acid matrix solution were mixed. Then, 0.3 µL of the mixture was spotted onto the surface. Once dry, the measurement was performed. Matrix-assisted laser desorption/ionization mass spectrometry experiment was conducted using an iMScope TRIO instrument (Shimadzu, Kyoto, Japan) equipped with a 1 kHz Nd:YAG laser (operating at a wavelength of 355 nm). Each pixel was irradiated 50 times at a repetition rate of 1 kHz. The laser power was set to 40.0 arbitrary units, and the detector voltage was set to 1.7 kV. The 2,5-Dihydroxybenzoic acid matrix solution was used as the internal standard for *m*/*z* calibration. Mass spectra were acquired in the positive ion detection mode. In the mass spectrometry experiment, the interval of data points were 10.10 pixel in the lateral and axial directions. After sample analysis, the spectral intensity was extracted using IMAGEREVEAL MS (Shimadzu, Kyoto, Japan). The active ingredients were identified by comparing the signals of ethanol extracts from the tested tablets with those of the above reference chemical compounds.

### 2.3. Animals and Diagnosis

We have studied 52 cats that visited our hospital, diagnosed as FCGS by the veterinarian, that started medication with Mutoral-I and II between April 2024 and October 2025, and completed medication between June 2024 and the end of December 2025. Of these 52 cases, 22 had already undergone tooth extraction before starting medication, and 30 had not undergone tooth extraction before or during the medication period. Besides these 52 cases, 20 cats diagnosed as FCGS at our hospital (7 cases with prior tooth extraction and 13 cases without tooth extraction) were excluded from our present study because they discontinued medication due to worsening physical signs caused by the onset of other diseases, or because continued follow-up could not be achieved depending on the owner’s circumstances. The above-mentioned rule made it difficult to include them in statistical analysis due to a lack of clinical data to be assessed in this study, because the absence of data at just one time point will exclude all other data from the analysis for that case at three different time points in repeated measures ANOVA.

In our present study, FCGS was diagnosed basically according to the methods described previously [3,11]. Briefly, clinical signs of gingivostomatitis (inflammation of the gums and oral mucosa, swelling, erythema, ptyalism, ulcers, and bleeding) were observed by the veterinarians. Erythema was assessed on a 3-point scale (0, 1.5 and 3) to indicate the inflammatory severity of gingivostomatitis, and ptyalism grade was also assessed on a 3-point scale (0, 1.5 and 3) to measure the severity of pain in the oral cavity. The scoring rules for each clinical sign are shown in Table 1. In addition, the owners were asked about the cat’s medical history (e.g., recent illness, exposure to infectious diseases, and the other clinical signs), and they were also interviewed to assess the level of three physical signs (appetite, activity level, and grooming behavior) at home, each of which was assessed on a 4-point scale (0–3). The scores for each physical sign are shown in Table 2.

All the studied cats were diagnosed as FCGS by the veterinarians in our hospital after comprehensive evaluation of oral mucosa observation, owner survey, physical signs and clinical laboratory parameters of the cats. Three physical signs (appetite, activity level, and grooming behavior), ptyalism, erythema level and weight were evaluated at their successive visits to our hospital: before the first administration, 1–2 weeks after the start of medication, 1–2 months after the start of medication, and just prior to the end of each medication period. The age, sex, and breed were recorded at their initial visit to our hospital.

Clinical samples for measuring clinical laboratory and hematological parameters (EDTA-containing whole blood, or plasma separated by centrifugation of heparinized whole blood) were collected at the four time points as described in the above paragraph. Circulating levels of plasma blood urea nitrogen (BUN), total blood protein (TP), albumin (ALB), and globulin (GLO) were measured using a DRI-CHEM4000V system (Fujifilm Corporation, Tokyo, Japan). Serum amyloid A (SAA) levels were measured using a Quick LASAY 101 system (Shima Laboratories, Inc., Tokyo, Japan). Hematocrit (HCT), white blood cell count (WBC), neutrophil count (NEU), and monocyte count (MON) were measured using a Procyte Dx (IDEXX Laboratories Inc., Westbrook, ME, USA).

Detailed information about FCGS treatment with Mutoral-I and II were provided to all of the cat owners at the initial consultation, including potential risks and benefits, estimated costs and treatment duration. Written informed consents were obtained in advance from all owners regarding the selected treatment after mutual agreement between the veterinarians and owners. Additionally, both parties confirmed that Mutoral-I and II were administered under optimal conditions to all cats as a standard of care and not as an experimental therapy. All data were obtained within the scope of normal veterinary practice and were appropriately anonymized. The studies were conducted in accordance with the local legislation and institutional requirements. The need for ethical review and approval was waived for these reasons.

### 2.4. Statistical Analysis

Age in months between tooth-extracted and unextracted groups was statistically compared using the Mann–Whitney nonparametric U test. Repeated measures ANOVA was used to determine any significant changes in the tested variables (weight, appetite, activity level, grooming behavior, ptyalism, erythema, HCT, WBC, NEU, MON, BUN, TP, ALB, GLO, and SAA biomarker levels) at the four time points: before the first treatment, 1–2 weeks after the start of treatment, 1–2 months after the start of treatment, and just prior to the completion of therapy. Repeated measures ANOVA, accounting for the measurement of physical signs or clinical laboratory parameters, surgical treatment group (tooth-extracted and unextracted), and interaction of measurement and group, was used to determine and compare significant changes in the tested variables at the four time points in the groups. Differences were considered significant at *p* < 0.05. StatView 5.0 (SAS Institute, Cary, NY, USA) was used for statistical analysis.

## 3. Results

### 3.1. Analysis of Changes in Physical Signs and Clinical Biomarker Levels

Of the 52 cats with FCGS entered in this study, 22 had undergone tooth extraction at any of the external veterinary hospitals before the start of our treatments, while the remaining 30 had no tooth extractions before or throughout our treatment period. No statistically significant difference was detected in the ages of the two groups (mean ± standard error of the mean; 91.2 ± 8.8 and 78.8 ± 7.3 weeks, respectively) by using Mann–Whitney nonparametric test. Tooth-extracted group (22 cases) contains 10 neutered males, 11 neutered females and 1 case of unknown. Unextracted group (30 cases) consisted of 21 neutered males, 7 neutered females, 1 intact male and 1 intact female. In terms of breed, tooth-extracted group consisted of one American Shorthair, one Russian Blue, and one Ragamuffin, with the remaining cases being mixed breeds, while unextracted group consisted of one Maine Coon and one Himalayan, with the remaining cats being mixed breeds.

**Figure 1:**
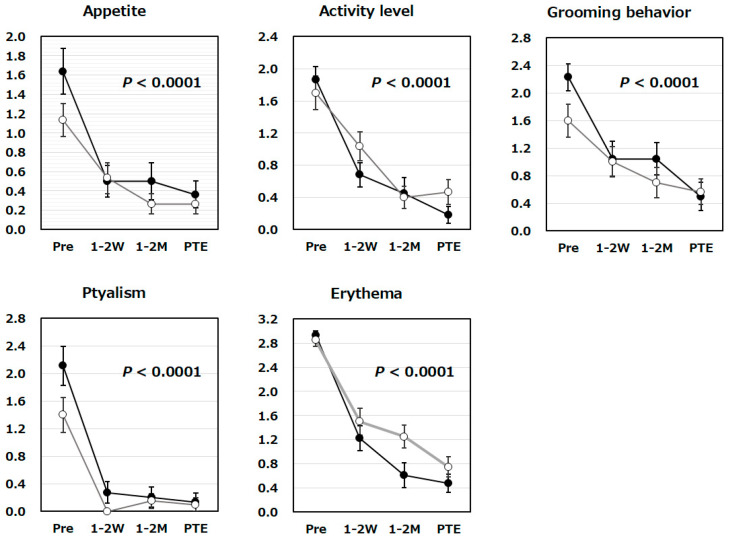
Changes in physical sign scores during Mutoral-I and II treatment period. Changes in scores for the studied physical signs were shown for tooth-extracted (●; N = 22) and unextracted groups (〇; N = 30) of the FCGS cats at the four time points: before administration (Pre), 1–2 weeks after the start of medication (1–2 W), 1–2 months after the start of medication (1–2 M), and just prior to the end of medication period (PTE). Time-dependent changes in all five scores were confirmed to be statistically significant (*p* < 0.0001). No interaction was observed between the case groups with and without prior tooth extraction in terms of each score decrease, and there was no statistically significant difference in the effects of the surgical treatments. Scorings for 5 physical signs were performed according to the criteria shown in Table 1 or Table 2. Symbols and vertical lines in the graph indicate mean values and standard errors, respectively.

Furthermore, tooth-extracted group received steroids in 15 cats (68.2%), antibiotics in 9 cats (40.9%), analgesics in 3 cats (13.6%), and immunosuppressants or cytokines in 3 patients (13.6%). Unextracted group had received steroids in 13 cats (43.3%), antibiotics in 13 cats (43.3%), analgesics in none (0.0%), and immunosuppressants or cytokines in 4 cats (13.3%). The studied cats were divided into two groups, tooth-extracted and unextracted, and the changes in 5 physical sign scores (rated on a scale from 0 to 3) of each group were analyzed at the four time points: immediately before the first administration of Mutoral-I, 1–2 weeks after the start of administration, 1–2 months after the start of administration, and just prior to the end of Mutoral-II administration period (Figure 1).

Statistically significant changes over time were observed for appetite, activity level, grooming behavior, ptyalism, and erythema scores (*p* < 0.0001). Significant decrease was observed over time for appetite, activity level or grooming behavior scores, and apparent trend toward a decrease was also detected for each of ptyalism and erythema scores (Figure 1). No interaction was observed between the cat groups with and without prior tooth extraction in terms of each score fluctuation by repeated measures ANOVA, demonstrating that there was no statistically significant difference in the effects of the surgical treatments.

**Figure 2:**
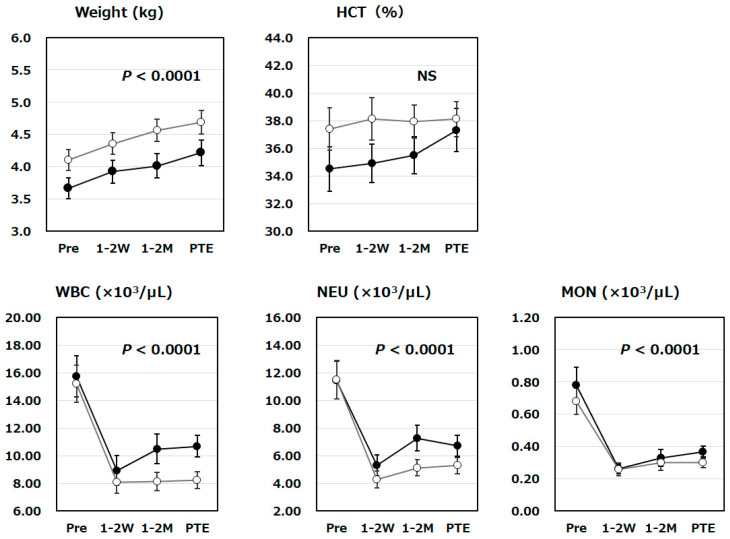
Changes in body weight and hematological biomarker levels during Mutoral-I and II treatment period. Time-dependent changes in body weight and hematological biomarker levels were shown for tooth-extracted (●; N = 22) and unextracted groups (〇; N = 30) of FCGS cats at the four time points: before administration (Pre), 1–2 weeks after the start of medication (1–2 W), 1–2 months after the start of medication (1–2 M), and just prior to the end of the medication period (PTE). Time-dependent changes in body weight, WBC, NEU and MON were all confirmed to be statistically significant (*p* < 0.0001), but no significance was observed in those of HCT (NS). No interaction was observed between the case groups with and without prior tooth extraction in terms of each parameter change, and there was no statistically significant difference in the effects of the surgical treatments. Symbols and vertical lines in the graph indicate mean values and standard errors, respectively. Abbreviations: HCT: hematocrit; WBC: white blood cell count; NEU: neutrophil count; MON: monocyte count; NS: Not significant.

We have analyzed the changes in body weight and hematological biomarker levels (HCT, WBC, NEU, and MON) at the four time points (Figure 2). A statistically significant increase in body weight was observed in both groups during these periods (*p* < 0.0001). Furthermore, hematological biomarkers (WBC, NEU and MON) decreased significantly with Mutoral-I and II treatments (*p* < 0.0001), regardless of whether tooth extractions were performed. No significant changes in HCT could be observed totally with Mutoral-I and II treatments, but only in the group with tooth extraction—a tendency for it to rise from low values before administration to the later period was observed, indicating slight recovery from temporary bleeding possibly due to tooth extraction, because the studied cats whose detected HCT was below 24% were under routine treatment for anemia. No interaction was observed between the case groups with and without prior tooth extraction in terms of the variations in any of these parameters by repeated measures ANOVA, revealing that there was no statistically significant difference in the effects of the surgical treatments on FCGS therapy with Mutoral-I and II.

**Figure 3:**
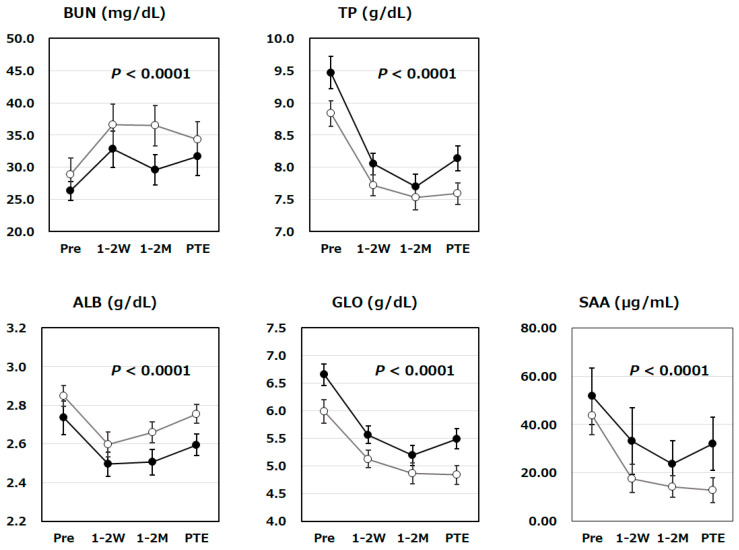
Changes in clinical laboratory biomarker levels during Mutoral-I and II treatment period. Changes in 5 clinical laboratory biomarker levels were shown for tooth-extracted (●; N = 22) and unextracted groups (〇; N = 30) of FCGS cats at the four time points: before administration (Pre), 1–2 weeks after the start of medication (1–2 W), 1–2 months after the start of medication (1–2 M), and just prior to the end of the medication period (PTE). Time-dependent changes in BUN, TP, ALB, GLO and SAA levels were all found to be statistically significant (*p* < 0.0001). No interaction was observed between the case groups with and without prior tooth extraction in terms of each parameter change, and there was no statistically significant difference in the effects of the surgical treatments. Symbols and vertical lines in the graph indicate mean values and standard errors, respectively. Abbreviations: BUN: blood urea nitrogen; TP: total blood protein; ALB: albumin; GLO: globulin; SAA: serum amyloid-A.

Furthermore, we have also investigated the changes in various clinical laboratory biomarker levels at the four time points (Figure 3). Circulating levels of TP, ALB, and GLO all decreased gradually along with Mutoral-I and II administration, regardless of whether tooth extractions were performed, and their decreases were statistically significant (*p* < 0.0001). BUN levels increased significantly with Mutoral-I and II administration, regardless of whether prior tooth extractions were carried out (*p* < 0.0001). In addition, SAA levels also significantly decreased along with Mutoral-I and II treatment (*p* < 0.0001). By using repeated measures ANOVA, no interaction was observed between the case groups with and without prior tooth extraction in terms of each clinical biomarker changes, and therefore, there was no statistically significant difference in the effects of the surgical treatments on the therapy with Mutoral-I and II.

### 3.2. Macroscopic Observation of Oral Mucosal Regions

**Figure 4:**
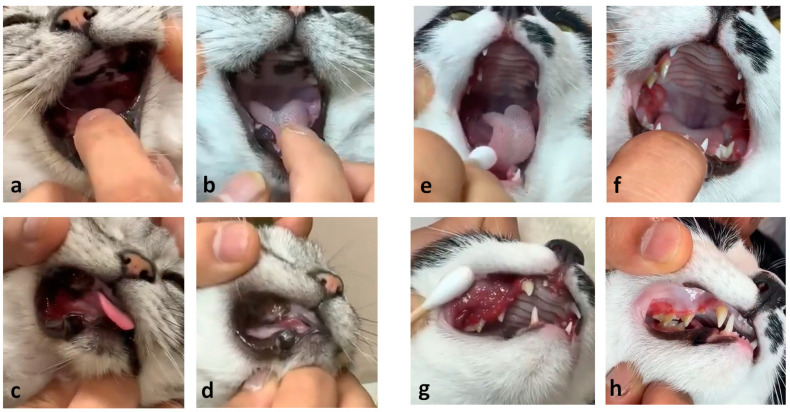
Clinical lesions in the studied cats with full tooth extraction (**left**) and no extraction (**right**). Mucosa of the caudal oral cavity (**top**) and maxillary gingiva (**bottom**) of the cats with tooth extraction (**a**–**d**) and no tooth extraction (**e**–**h**) before and just prior to the end of Mutoral-I and II administration. In the cat with full tooth extraction, erythema was observed in the oral cavity (**a**) or the maxillary gingiva (**c**), but normalized by the end of Mutoral-I and II medication period (**b**,**d**, respectively). In the cat with no tooth extraction, erythema was initially observed in the oral cavity (**e**) and maxillary gingiva (**g**), but normalized by the end of the medication period (**f**,**h**, respectively).

Among the cats with FCGS included in this study, one case each from tooth-extracted and unextracted groups, for which video images could be acquired, has been selected. Macroscopic observations of their oral cavity and maxillary gingiva by the veterinarians before and just prior to the end of Mutoral-I and II treatment are shown in Figure 4. In the tooth-extracted case (American Shorthair, neutered female, with full tooth extraction, 91 weeks-aged at the start of Mutoral-I and II treatment), inflammatory erythema was observed in the oral cavity and maxillary gingiva before treatment (Figure 4a,c), but improved until the end of the treatment period (Figure 4b,d, respectively). In the unextracted case (mixed breed, neutered male, no tooth extraction, 12 weeks-aged at the start of treatment), erythema was observed in the oral cavity and maxillary gingiva before treatment (Figure 4e,g), but apparently normalized by the treatment (Figure 4f,h, respectively). Both cases received oral administration of Mutoral-I and II according to the standard administration method and schedule as described above. The former case, who underwent tooth extraction, visited our hospital because the clinical signs characteristic of FCGS persisted and did not improve despite full extraction of the teeth.

### 3.3. Mass Spectrometric Analysis of Active Ingredients Using Standard Substances

**Figure 5:**
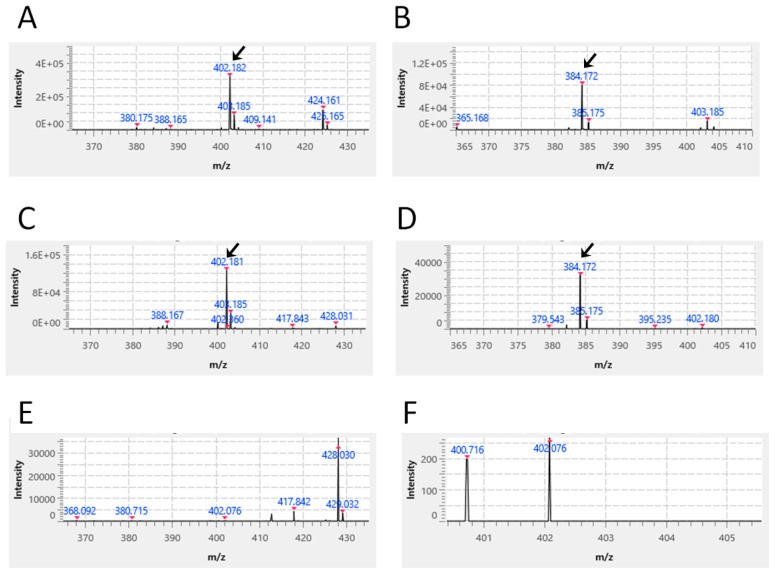
Mass spectrometric detection of Moxifloxacin. Expected signals ([M+H] + *m*/*z*: 402.18 fragmented into 384.17) were detected using Moxifloxacin solution as a standard (arrows in **A**,**B**, respectively), and the equivalent signal was apparently observed in the ethanol extracts of Mutoral-I (arrows in **C**,**D**, respectively). No similar signal could be detected in the extracts of Mutian-II (**E**,**F**).

Using a standard solution (100 μg/mL) of Moxifloxacin (chemical formula: C_21_H_24_FN_3_O_4_; molecular weight: 401.43; exact mass: 401.175), prepared using commercially available reagents, the expected signal ([M+H] + *m*/*z*: 402.18 fragmented into 384.17) was detected by mass spectrometry (arrows in Figure 5A,B, respectively). Next, using the extract obtained by thoroughly crushing one Mutoral-I tablet in 2 mL of ethanol, the same signal ([M+H] + *m*/*z*: 402.18 fragmented into 384.17) was detected by the same procedure (arrows in Figure 5C,D, respectively). However, when using the extract obtained by thoroughly crushing one Mutoral-II tablet in 2 mL of ethanol, the corresponding signal was not detected (Figure 5E,F). These data demonstrated that Mutoral-I tablets contain Moxifloxacin as an active ingredient.

**Figure 6:**
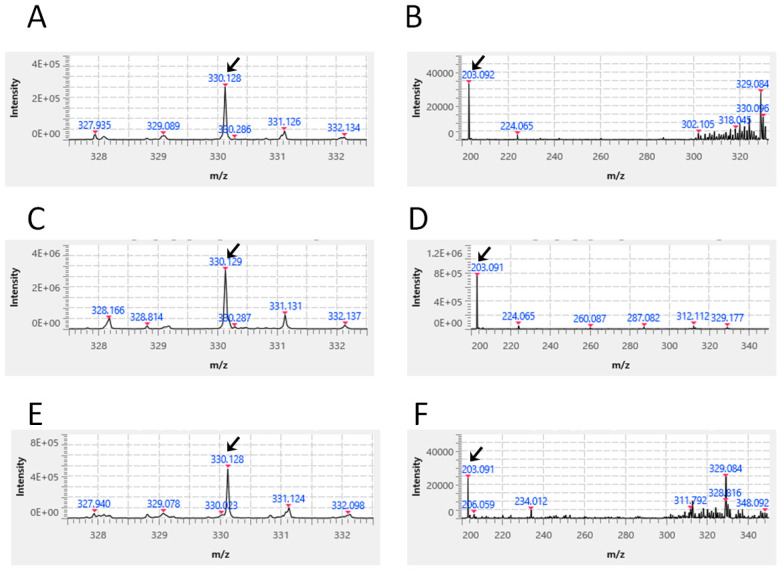
Mass spectrometric detection of Molnupiravir. Expected signal ([M+H] + *m*/*z*: 330.13 fragmented into 203.09) was detected using Molnupiravir solution as a standard (arrows in **A**,**B**, respectively). The equivalent signals were apparently observed in the ethanol extracts of Mutoral-I (arrows in **C**,**D**, respectively) and Mutoral-II tablets (arrows in **E**,**F**, respectively).

When a standard solution (200 μg/mL) of Molnupiravir (chemical formula: C_13_H_19_N_3_O_7_; molecular weight: 329.309; exact Mass: 329.122) prepared using commercially available reagents was used, a signal expected from the compound information ([M+H] + *m*/*z*: 330.13 fragmented into 203.09) was detected by mass spectrometry (arrows in Figure 6A,B, respectively). Next, when an extract obtained by thoroughly crushing one Mutoral-I tablet in 2 mL of ethanol solvent was used, a signal equivalent to the above ([M+H] + *m*/*z*: 330.13 fragmented into 203.09) was detected (arrows in Figure 6C,D, respectively). Furthermore, when the extract obtained by thoroughly crushing one Mutoral-II tablet in 2 mL of ethanol was used, the identical signal ([M+H] + *m*/*z*: 330.13 fragmented into 203.09) was detected (Figure 6E,F, respectively). These mass spectrometry results showed that the Mutoral-I formulation contains Moxifloxacin as the active ingredient, and that Molnupiravir could be detected as the active ingredient in both Mutoral-I and II.

## 4. Discussion

As almost all the FCGS-affected cats will exhibit moderate to severe periodontitis and tooth resorption, the standard treatment for FCGS is surgical extraction of premolars and molars, or even entire dental extractions. Periodontitis is a substantial inflammatory burden on the oral mucosa and the immune system. Therefore, extraction of teeth will effectively reduce a portion of the chronic inflammatory burden, allowing for a subpopulation of patients to achieve a significant improvement or even a cure. However, only a limited number of cases demonstrate clear symptom improvement with tooth extraction alone, and adjunctive medical treatment with immunosuppressants, analgesics, and antibiotics is required [3,4]. Approximately 30% of FCGS cases do not respond to complete tooth extraction, and the other potential treatments also show insufficient response. Due to the decline in quality of life, some cat owners may choose humane euthanasia [1,3]. Furthermore, spontaneous recovery from FCGS has not been reported, and any efficient solution is strongly needed.

One possible explanation for the disease improvement exhibited by tooth extraction is that surgical removal of the inflammatory mucosal tissues in the oral cavity may reduce chronic antigenic stimulation caused by bacterial or viral infection in the affected area [7,8,9]. While the underlying etiology of FCGS remains unknown, several infectious pathogens have been suggested to be associated with the onset and severity of the disease, with FCV infection being a particularly strong suspect. Specifically, comparative studies of FCV gene detection rates and oral biopsy studies have suggested a link between FCV infection and FCGS [7,8,9]. However, the lack of correlation between FCV RNA levels in FCV-positive cats diagnosed with FCGS and disease severity has cast doubt on the hypothesis that FCV infection alone is the primary cause of FCGS [4]. Furthermore, immunohistochemical analysis previously performed on FCGS cats with the FeLV antigen present in the epithelium revealed inflammatory infiltrates in 30.8% of cats with FCGS. However, the FCV antigen was not detected in the lesions. This suggests that a correlation between FCGS and FCV infection may be present, and that FeLV may play a role as the causative agent of the lesions [12]. Thus, for FCGS treatments, we should consider not only FCV but also other viral infections such as FHV-1, FIV, and FeLV. Furthermore, effectiveness of surgical tooth extraction for FCGS treatment has been limited, and therefore, various alternative treatments (e.g., cyclosporine, feline interferon-ω, cannabidiol, omega-3 polyunsaturated fatty acids, and their combinations) have been clinically evaluated. However, none of these treatments have been clearly demonstrated to be effective, and they have not yet been adopted as standard treatments in the current veterinary practice [13,14,15,16,17,18].

Here, we hypothesized that combining a broad-spectrum antibacterial agent with an antiviral agent effective against multiple RNA viruses might be an effective treatment for FCGS, as one way to fundamentally resolve the current challenges in FCGS treatment described above. We therefore utilized a stepwise combination therapy using Mutoral-I and Mutoral-II, which share a similar therapeutic concept. Furthermore, because the manufacturer’s information regarding the active ingredients of both formulations was extremely limited, we have performed mass spectrometric analysis for their active ingredients ourselves. It was demonstrated that the active ingredients of Mutoral-I consist of Moxifloxacin as the antibacterial and Molnupiravir as the antiviral agent, while the active ingredient of Mutoral-II is Molnupiravir alone (Figure 5 and Figure 6). The former, Moxifloxacin, is an orally administrable quinolone-body compound with a broad antibacterial spectrum. It has been shown to be particularly effective against some periodontal pathogens such as *Porphyromonas gingivalis* and *Fusobacterium nucleatum* with a very short acting period, and its safety has been confirmed for use in animals, including cats [19,20]. Molnupiravir, meanwhile, is an orally administered prodrug (EIDD2801) of N4-hydroxycytidine (EIDD1931), which was developed as a therapeutic drug against COVID-19. It has been shown to exhibit antiviral activity against feline coronavirus (FCoV) [21,22]. This drug has been approved in various countries for the treatment of human COVID-19 patients, and we have already investigated its clear therapeutic effect in treating the cats with feline infectious peritonitis in our hospital [23].

There has been no previous report demonstrating the anti-FCV activity of Molnupiravir, but a similar cytidine-like nucleoside analog, 2’-C-methylcytidine (2CMC), has been shown to inhibit FCV replication at low micromolar concentrations, both of which can inhibit the viral RNA-dependent RNA polymerase [24]. The recent study has shown 2CMC exhibits anti-norovirus and anti-rotavirus activity, similarly to the other previously reported anti-norovirus and anti-rotavirus drugs, such as 7-deaza-2’-C-methyladenosine, nitazoxanide, favipiravir, and dasabuvir. In addition, Molnupiravir, a COVID-19 therapeutic, and its active metabolite, EIDD1931, have also been shown to show anti-norovirus and anti-rotavirus activity [25]. Norovirus, the well-known leading cause of gastrointestinal diarrhea, is a member of the *Caliciviridae* family, in which FCV is classified also. Furthermore, Molnupiravir is basically thought to exhibit broad-spectrum antiviral activity due to its structural characteristics as a pyrimidine nucleoside inhibitor [26]. Therefore, the authors considered the above previous results comprehensively and prospectively, leading to the hypothesis that Molnupiravir is expected to have a potential antiviral effect against FCV as with previously reported cases of drug repurposing.

In order to examine the efficacy of these antibacterial and antiviral combinations in treating the 52 cats with FCGS, we have measured their physical signs, hematological, and clinical laboratory indicators as quantitative biomarkers and statistically analyzed the fluctuations of their levels over time. Three physical signs (appetite, activity level, and grooming behavior) scored by the owner evaluation in our present study are known to be used to calculate the Stomatitis Disease Activity Index (SDAI), a previously established method for assessing the severity of FCGS [13]. Significant improvements in these physical signs were observed during Mutoral-I and II treatments. Additionally, a statistically significant reduction in the score for erythema and proliferation, also used to calculate the SDAI, was observed over time during treatment (Figure 1). Furthermore, ptyalism, observed as excessive salivation commonly induced by oral diseases such as stomatitis, whose scores were quantified by the veterinarians in order to measure oral pain and discomfort of the cats, has also tended to decline over time during Muroral-I and II treatments, suggesting dramatic improvements in their oral inflammation (Figure 1). Body weight, one of the factors used to calculate the SDAI, also showed a clear tendency for increase during Mutoral-I and II medication (Figure 2). Thus, statistically significant improvements were observed in all SDAI components, confirming the effectiveness of Mutoral-I and II medication on FCGS treatments. For calculating the SDAI score, it is necessary for veterinarians to visually examine the lesions in the oral cavity. This would require, however, the veterinarian’s deep knowledge and long-standing clinical experience in FCGS syndrome, making absolute assessment extremely difficult [3].

Furthermore, statistical analysis of various parameters requires a certain number of cases, but most of the previous clinical studies have included fewer than 50 cases, making the validity of statistical analysis questionable [27]. For resolution of these issues, we have evaluated the usefulness of our new therapeutics by dividing 50 or more cases of FCGS patients into the first group with prior tooth extraction, an already-existing surgical procedure, and the second group without tooth extraction, to be used as clinical subjects under evaluation. In this study, we conducted statistical significance tests for time-dependent changes in physical signs (scored by the owners or veterinarians) and clinical laboratory biomarker levels (quantitative parameters determined by the automated equipment in our hospital) by using repeated measures ANOVA.

Some previous studies have already revealed a positive correlation between hematological parameters such as WBC, NEU, and MON counts and immune-induced inflammatory responses in the oral mucosa of FCGS, reasonably suggesting that the disease is not necessarily limited to the oral cavity but is a systemic condition [28,29]. A recent study has already reported that statistically significant increases in all three hematological parameters were observed in 34 cats with viral gingivostomatitis compared with 21 cats with nonviral gingivostomatitis, but no significant differences were detected in other hematological parameters (e.g., lymphocyte count, red blood cell count, or platelet count) between the two groups [30]. Consistent with these findings, WBC, NEU, and MON counts are efficient indicators suitable for monitoring the host immune response to viral infection, which is believed to be the cause of FCGS. Our current study has also suggested their usefulness in assessing FCGS treatment efficacy (Figure 2).

SAA, a well-known clinical laboratory indicator frequently used as a biomarker of inflammatory responses, has also been reported to be elevated in FCGS-affected cats [31]. Hyperglobulinemia, along with feline infectious peritonitis, has also been frequently observed in the cats with FCGS, indicating their enhanced immune responses [32,33]. However, previous evaluations of these biomarkers have only focused on statistical comparison of clinical parameters between healthy and FCGS cats. Our present study is the first report to investigate time-dependent changes in clinical biomarkers during FCGS treatment, especially under successive medication, and to demonstrate their statistical significance (Figure 2 and Figure 3). Furthermore, this study also examined whether statistically significant differences were detected in the fluctuations of these biomarkers between the tooth-extracted and unextracted groups. As a result, no statistically significant differences were detected in the changes in any of the biomarkers between these two groups (Figure 1, Figure 2 and Figure 3). This result has actively suggested that such a stepwise combination therapy with antibacterial and antiviral agents would be effective in treating FCGS, even without prior tooth extraction. It is widely understood that general surgical tooth extraction procedures usually require anesthesia for cats, which poses a certain risk to life and should be avoided if possible. The tooth extraction procedure, as well as the decision of whether to perform partial or complete tooth extraction, is highly dependent on the veterinarian’s surgical skill, and such a surgical approach has been suggested as not always leading to apparent improvement in FCGS. The results obtained by our present study are expected to lead to the development of safer FCGS treatments that do not require the risk to life caused by anesthesia.

Limitations of our present study include the fact that it is unclear whether the antibacterial agent, Moxifloxacin, may be effective against the pathogenic bacteria in the oral cavity of FCGS-affected cats, and also that it has not been demonstrated yet at this time whether the antiviral agent, Molnupiravir, has an apparent antiviral activity against FCV, which is possibly indicated to be the primary cause of FCGS, or the other viruses (FeLV, FIV, FHV-1) that have also been implicated in the onset of FCGS. Given the promising results obtained in our current prospective approach, it would be expected that the antibacterial and antiviral effects of these compounds will be confirmed in in vitro or in vivo experiments in any reverse-translational studies as soon as possible.

A second limitation of our current study is that while the presence of the above two active ingredients in Mutoral-I and Mutoral-II formulations was confirmed, their exact amounts have not been determined and are based solely on the manufacturer’s information. More scientific verification of this information is desirable. Therefore, we are planning to measure the amounts of active ingredients exactly in these tablets and will report their information successfully later than our current study. It is also understood, however, since such an experimental trial may be fairly long-term, we have decided to disclose the identification of active ingredients in our present report at this stage. Furthermore, because the extremely expanded use of antibacterial and antiviral drugs carries the potential risk of encouraging the emergence of resistant infectious strains, it is essential that they are always used under the veterinarian’s supervision. Additionally, it is also necessary that some educational activities continue to be carried out on social media and the other platforms, for the pet owners and the animal shelter organizations, to prevent individual owners from purchasing imported drugs and using them to treat the cats with FCGS by themselves.

## 5. Conclusions

This study is the first report to describe the efficacy of stepwise combination therapy with Moxifloxacin as an antibacterial and Molnupiravir as an antiviral agent in cats with FCGS. Their therapeutic effect on FCGS-affected cats could be apparently confirmed by determining the levels of multiple hematological parameters (WBC, NEU, and MON) and usual clinical laboratory biomarkers (especially SAA and GLO), and could also be easily assessed using their physical signs (appetite, activity level, grooming behavior, ptyalism, or erythema scores), which are components of the SDAI score. Because the efficacy of this treatment was not distinctive between the cases in which surgical tooth extraction had been performed and those in which it was not, this combination medication can be understood to be preferable as compared with tooth extraction against FCGS. We believe that this treatment for FCGS will be widely adopted for veterinary care after any sufficient clinical trials completed in the future.

## Figures and Tables

**Table 1:** Scoring for erythema and ptyalism of the studied cats by veterinarians.

Target	Score	Criteria
Erythema	3	Severe
	1.5	Moderate
	0	None
Ptyalism	3	Severe
	1.5	Moderate
	0	None

**Table 2:** Scoring for physical signs of the studied cats by owner evaluation.

Target	Score	Criteria
Appetite	3	Eats only pureed food, or only when hand fed
	2	Eats wet food, cannot eat dry food
	1	Eating wet and dry food, but less than normal amount
	0	Eating normally
Activity level	3	No interest in people or other pets, spends most of time sleeping
	2	Low activity level, but will play occasionally when engaged by people or other pets
	1	Plays spontaneously, but not frequently
	0	Normal activity level (playful and active)
Grooming behavior	3	Will not groom
	2	Grooms occasionally but not at ‘pre-illness’ level
	1	Grooming excessively
	0	Grooming normally

## Data Availability

The data presented in this study are available on request from the corresponding author due to the protection of personal information for the owners and their cats.

## References

[1] Healey K.A., Dawson S., Burrow R., Cripps P., Gaskell C.J., Hart C.A., Pinchbeck G.L., Radford A.D., Gaskell R.M. (2007). Prevalence of feline chronic gingivo-stomatitis in first opinion veterinary practice. J. Feline Med. Surg..

[2] Kim D.H., Kwak H.H., Woo H.M. (2023). Prevalence of feline chronic gingivostomatitis in feral cats and its risk factors. J. Feline Med. Surg..

[3] Soltero-Rivera M., Goldschmidt S., Arzi B. (2023). Feline chronic gingivostomatitis current concepts in clinical management. J. Feline Med. Surg..

[4] Druet I., Hennet P. (2017). Relationship between Feline calicivirus Load, Oral Lesions, and Outcome in Feline Chronic Gingivostomatitis (Caudal Stomatitis): Retrospective Study in 104 Cats. Front. Vet. Sci..

[5] Sánchez-Vallejo M., Vélez-Velásquez P., Correa-Valencia N.M. (2025). Feline chronic gingivostomatitis: A thorough systematic review of associated factors. J. Feline Med. Surg..

[6] Jennings M.W., Lewis J.R., Soltero-Rivera M.M., Brown D.C., Reiter A.M. (2015). Effect of tooth extraction on stomatitis in cats: 95 cases (2000–2013). J. Am. Vet. Med. Assoc..

[7] Fried W.A., Soltero-Rivera M., Ramesh A., Lommer M.J., Arzi B., DeRisi J.L., Horst J.A. (2021). Use of unbiased metagenomic and transcriptomic analyses to investigate the association between feline calicivirus and feline chronic gingivostomatitis in domestic cats. Am. J. Vet. Res..

[8] Nakanishi H., Furuya M., Soma T., Hayashiuchi Y., Yoshiuchi R., Matsubayashi M., Tani H., Sasai K. (2019). Prevalence of microorganisms associated with feline gingivostomatitis. J. Feline Med. Surg..

[9] Hofmann-Lehmann R., Hosie M.J., Hartmann K., Egberink H., Truyen U., Tasker S., Belák S., Boucraut-Baralon C., Frymus T., Lloret A. (2022). Calicivirus Infection in Cats. Viruses.

[10] Wogan L. Unproven Treatment for Oral Disease in Cats Surfaces in US. https://news.vin.com/default.aspx?pid=210&Id=12099794&f5=1#update.

[11] Lobprise H., St Denis K., Anderson J.G., Hoyer N., Fiani N., Yaroslav J. (2025). 2025 FelineVMA feline oral health and dental care guidelines. J. Feline Med. Surg..

[12] Rolim V.M., Pavarini S.P., Campos F.S., Pignone V., Faraco C., Muccillo M.S., Roehe P.M., da Costa F.V., Driemeier D. (2017). Clinical, pathological, immunohistochemical and molecular characterization of feline chronic gingivostomatitis. J. Feline Med. Surg..

[13] Lommer M.J. (2013). Efficacy of cyclosporine for chronic, refractory stomatitis in cats: A randomized, placebo-controlled, double-blinded clinical study. J. Vet. Dent..

[14] Hennet P.R., Camy G.A., McGahie D.M., Albouy M.V. (2011). Comparative efficacy of a recombinant feline interferon omega in refractory cases of calicivirus-positive cats with caudal stomatitis: A randomised, multi-centre, controlled, double-blind study in 39 cats. J. Feline Med. Surg..

[15] Matsumoto H., Teshima T., Iizuka Y., Sakusabe A., Takahashi D., Amimoto A., Koyama H. (2018). Evaluation of the efficacy of the subcutaneous low recombinant feline interferon-omega administration protocol for feline chronic gingivitis-stomatitis in feline calicivirus-positive cats. Res. Vet. Sci..

[16] Coelho J.C., Duarte N., Bento da Silva A., Bronze M.D.R., Mestrinho L.A. (2023). Placebo-Controlled Trial of Daily Oral Cannabidiol as Adjunctive Treatment for Cats with Chronic Gingivostomatitis. Animals.

[17] Sukho P., Ploypetch S., Satthathum C., Prompiram P., Chakritbudsabong W. (2025). Efficacy and safety of omega-3-enriched lickable treats as adjunctive therapy for feline chronic gingivostomatitis: A randomized controlled trial. Vet. World.

[18] Choe K.H., Jang K., Kim S.E., Jo H.M. (2025). Long-term efficacy of cyclosporine and interferon-ω in feline chronic gingivostomatitis: Insights from SDAI scores. BMC Vet. Res..

[19] Milazzo I., Blandino G., Musumeci R., Nicoletti G., Lo Bue A.M., Speciale A. (2002). Antibacterial activity of moxifloxacin against periodontal anaerobic pathogens involved in systemic infections. Int. J. Antimicrob. Agents.

[20] Meena M., Prajapat A., Deori N., Gurjar T., Patel P., Saini S. (2019). Moxifloxacin and its therapeutic uses in animals: An overview. J. Entomol. Zool. Stud..

[21] Wahl A., Gralinski L.E., Johnson C.E., Yao W., Kovarova M., Dinnon K.H., Liu H., Madden V.J., Krzystek H.M., De C. (2021). SARS-CoV-2 infection is effectively treated and prevented by EIDD-2801. Nature.

[22] Cook S.E., Vogel H., Castillo D., Olsen M., Pedersen N., Murphy B.G. (2022). Investigation of monotherapy and combined anticoronaviral therapies against feline coronavirus serotype II in vitro. J. Feline Med. Surg..

[23] Katayama M., Uemura Y., Katori D. (2024). Effect of Nucleic Acid Analog Administration on Fluctuations in the Albumin-to-Globulin Ratio in Cats with Feline Infectious Peritonitis. Animals.

[24] Fumian T.M., Tuipulotu D.E., Netzler N.E., Lun J.H., Russo A.G., Yan G.J.H., White P.A. (2018). Potential Therapeutic Agents for Feline Calicivirus Infection. Viruses.

[25] Santos-Ferreira N., Van Dycke J., Chiu W., Neyts J., Matthijnssens J., Rocha-Pereira J. (2024). Molnupiravir inhibits human norovirus and rotavirus replication in 3D human intestinal enteroids. Antivir. Res..

[26] Tian L., Qiang T., Liang C., Ren X., Jia M., Zhang J., Li J., Wan M., YuWen X., Li H. (2021). RNA-dependent RNA polymerase (RdRp) inhibitors: The current landscape and repurposing for the COVID-19 pandemic. Eur. J. Med. Chem..

[27] Winer J.N., Arzi B., Verstraete F.J. (2016). Therapeutic Management of Feline Chronic Gingivostomatitis: A Systematic Review of the Literature. Front. Vet. Sci..

[28] Vapniarsky N., Simpson D.L., Arzi B., Taechangam N., Walker N.J., Garrity C., Bulkeley E., Borjesson D.L. (2020). Histological, Immunological, and Genetic Analysis of Feline Chronic Gingivostomatitis. Front. Vet. Sci..

[29] Lopes R.S., Carvalho P.P., Pires M.A., Rodrigues-Santos P., Costa E., Requicha J.F. (2025). Local and systemic immunological response in feline chronic gingivostomatitis: A critical review. Front. Immunol..

[30] Korkmaz S.G., Ok M. (2025). Assessment of Selected Endothelial Damage Biomarkers in the Determination of Endothelial Damage in Cats with Gingivostomatitis. Vet. Med. Sci..

[31] Oikonomidis I.L., Kavarnos I., Papadimitriou S., Ceron J.J., Kouki M., Adamama-Moraitou K.K., Soubasis N. (2025). Serum Amyloid A and Haptoglobin as Markers in Cats with Gingivitis-Preliminary Study. Vet. Med. Sci..

[32] Harley R., Gruffydd-Jones T.J., Day M.J. (2003). Salivary and serum immunoglobulin levels in cats with chronic gingivostomatitis. Vet. Rec..

[33] Arzi B., Clark K.C., Sundaram A., Spriet M., Verstraete F.J.M., Walker N.J., Loscar M.R., Fazel N., Murphy W.J., Vapniarsky N. (2017). Therapeutic Efficacy of Fresh, Allogeneic Mesenchymal Stem Cells for Severe Refractory Feline Chronic Gingivostomatitis. Stem Cells Transl. Med..

